# Supplementary material for: Potential cross-species correlations in social hierarchy and memory between mice and young children
Source: Commun Biol. 2022 Mar 14;5:230. doi: 10.1038/s42003-022-03173-7 (PMC8921227; doi:10.1038/s42003-022-03173-7)
Supplement: Supplementary file 2 — Supplementary information [file 42003_2022_3173_MOESM2_ESM.pdf]

**Supplementary Figure 1**

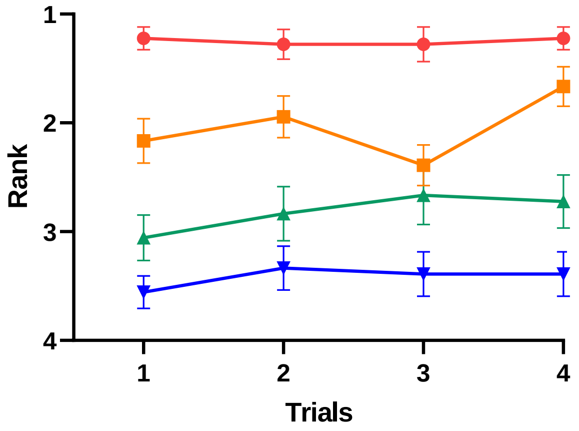


**Supplementary Figure 1. Summary of social ranks.** Summary of the social ranks of the weanling mice as determined by the tube test over four trials (n = 14 cages). Error bars = SEM.

**Supplementary Figure 2**

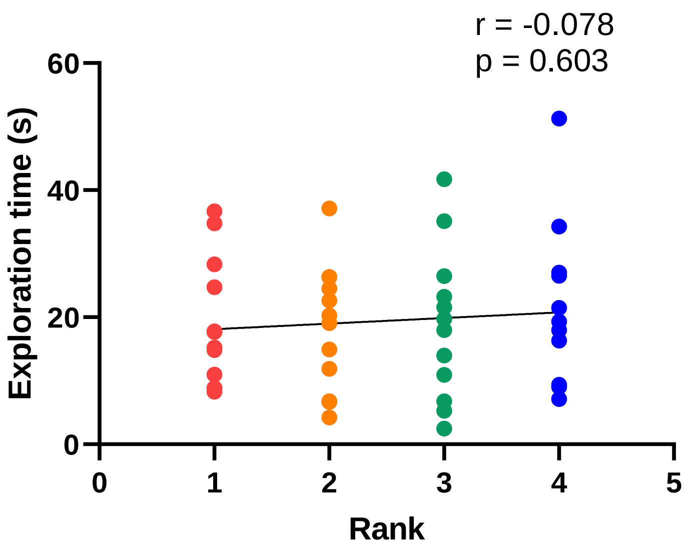


**Supplementary Figure 2. There was no significant correlation between social rank and exploratory behavior.** The correlation between rank and exploration time in the novelty investigation test (Spearman correlation, n = 12 cages).

## Supplementary Figure 3

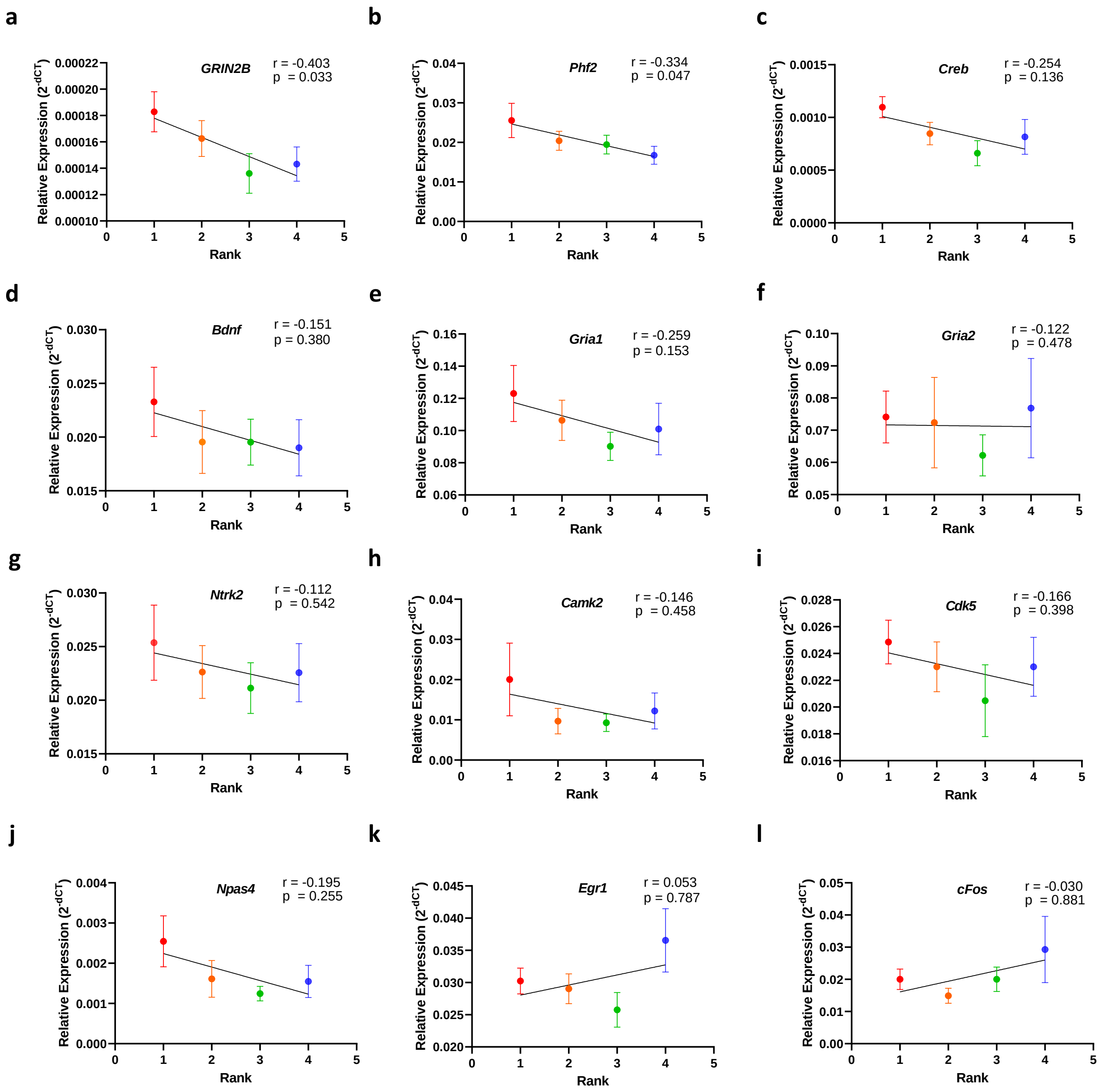

**Supplementary Figure 3. Dominant weanling mice tended to have a higher expression of several memory-related genes than subordinate weanling mice. a** The correlation between social rank and *Grin2b* expression (n = 7 cages). **b** The correlation between social rank and *Phf2* expression (n = 9 cages). **c** The correlation between social rank and *Creb* expression (n = 9 cages). **d** The correlation between social rank and *Bdnf* expression (n = 9 cages). **e** The correlation between social rank and *Gria1* expression (n = 8 cages). **f** The correlation between social rank and *Gria2* expression (n = 9 cages). **g** The correlation between social rank and *Ntrk2* expression (n = 8 cages). **h** The correlation between social rank and *Camk2* expression (n = 7 cages). **i** The correlation between social rank and *Cdk5* expression (n = 7 cages). **j** The correlation between social rank and *Npas4* expression (n = 9 cages). **k** The correlation between social rank and *Egr1* expression (n = 7 cages). **l** The correlation between social rank and c*Fos* expression (n = 7 cages). Spearman correlation. Error bars = SEM.

**Supplementary Figure 4**

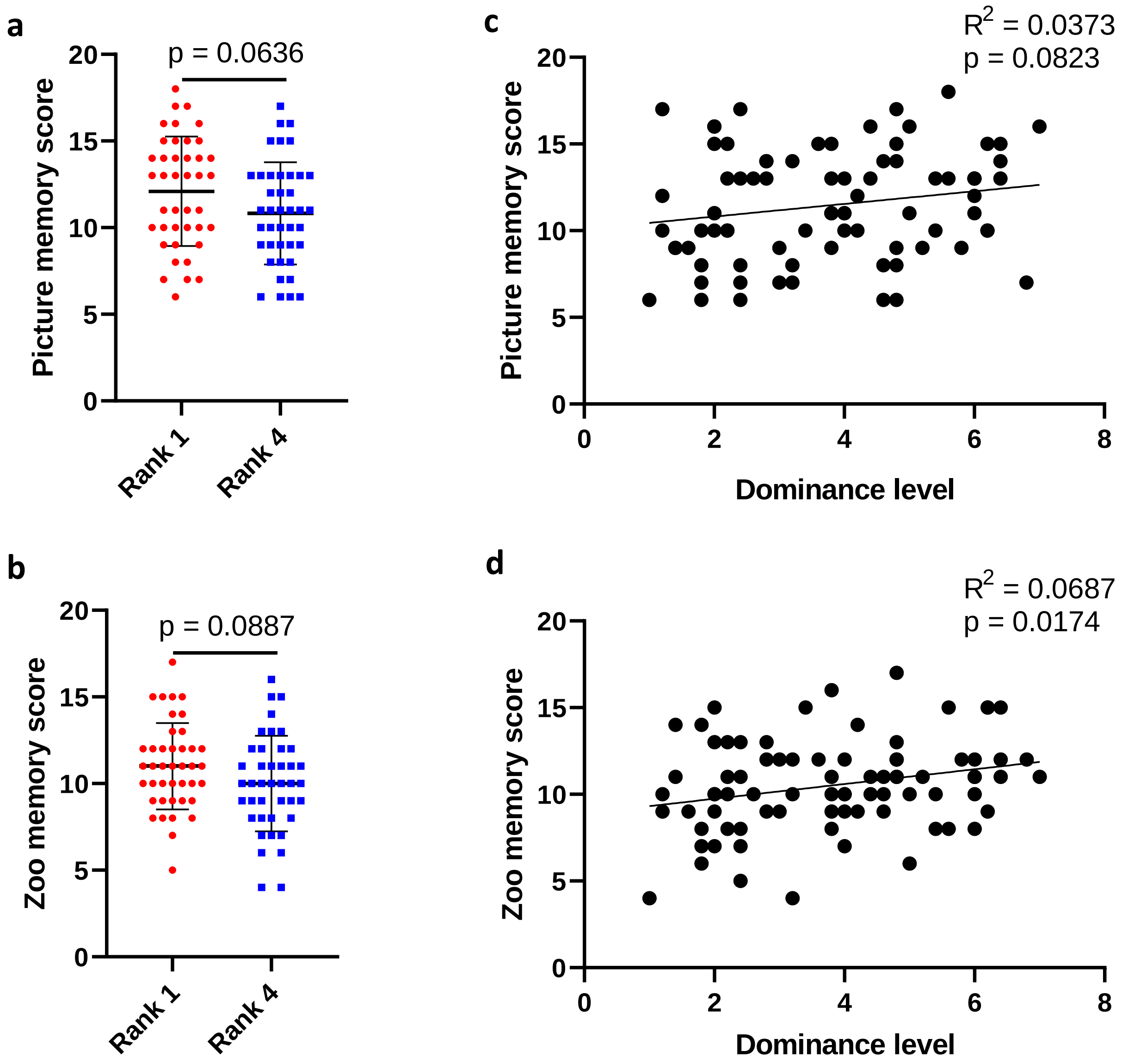


**Supplementary Figure 4. Children with higher rank or dominance levels tended to better memory. a** The picture memory test scores for 1st- and 4th-rank children (unpaired t test, n = 82 pairs). **b** The zoo memory test scores for 1st- and 4th-rank children (unpaired t test, n = 82). **c** The correlation between picture memory scores and dominance levels (Pearson correlation, n = 82). **d** The correlation between zoo memory scores and dominance levels (Pearson correlation, n = 82). Error bars = SEM.

**Supplementary Table 1. The consistency between social rank and expression level of memory-related genes**

| Gene | Rank Pairing | | | | | | Mean | Z score | P value |
|---|---|---|---|---|---|---|---|---|---|
| | 1:4 | 1:3 | 2:4 | 1:2 | 2:3 | 3:4 | | | |
| *Phf2* | 100.000 | 77.778 | 77.778 | 77.778 | 55.556 | 77.778 | 77.778 | 4.910 | <0.001 |
| *Creb* | 66.667 | 88.889 | 66.667 | 66.667 | 66.667 | 33.333 | 69.048 | 2.280 | 0.023 |
| *Grin2b* | 71.429 | 71.429 | 71.429 | 57.143 | 71.429 | 57.143 | 66.667 | 2.291 | 0.022 |
| *Gria1* | 62.500 | 75.000 | 62.500 | 75.000 | 75.000 | 37.500 | 64.583 | 2.113 | 0.035 |
| *CamkIIa* | 71.429 | 57.143 | 42.857 | 85.714 | 71.429 | 57.143 | 64.286 | 1.932 | 0.053 |
| *Gria2* | 66.667 | 77.778 | 55.556 | 55.556 | 66.667 | 44.444 | 61.905 | 1.675 | 0.094 |
| *Bdnf* | 55.556 | 77.778 | 44.444 | 66.667 | 44.444 | 55.556 | 61.905 | 1.101 | 0.271 |
| *Npas* | 55.556 | 55.556 | 44.444 | 55.556 | 44.444 | 44.444 | 57.143 | 0.000 | >0.999 |
| *Cdk5* | 71.429 | 71.429 | 42.857 | 57.143 | 42.857 | 28.571 | 52.381 | 0.309 | 0.758 |
| *Egr1* | 28.571 | 57.143 | 14.286 | 42.857 | 57.143 | 28.571 | 45.238 | -1.589 | 0.112 |
| *Trkb* | 50.000 | 75.000 | 25.000 | 37.500 | 62.500 | 37.500 | 45.238 | -0.289 | 0.773 |
| *Cfos* | 57.143 | 57.143 | 42.857 | 57.143 | 14.286 | 42.857 | 38.095 | -0.620 | 0.535 |

*Note. The values indicate the percentage of higher rank mice with higher gene expression than lower rank mice.*

**Supplementary Table 2. Regression analysis of the mediation effect: children memory→prosocial strategy→social dominance (n=175)**

| | Prosocial strategy | Social dominance | | |
|---|---|---|---|---|
| | Model 1 | Model 2 | Model 3 | Model 4 |
| | $\beta$ | $\beta$ | $\beta$ | $\beta$ |
| **Memory ability** | 0.46*** | 0.52*** | | 0.33*** |
| **Prosocial strategy** | | | 0.56*** | 0.40*** |
| $\boldsymbol{R^2}$ | 0.21 | 0.27 | 0.31 | 0.39 |
| ***Adj*** $\boldsymbol{R^2}$ | 0.20 | 0.26 | 0.30 | 0.39 |
| ***F*** | 45.09*** | 62.29*** | 76.82*** | 55.99*** |
| ***df*** | 1.173 | 1.173 | 1.173 | 2.172 |

***$p < 0.001$.

**Supplementary Table 3. Two-way ANOVA of FN400 by social status (1st- and 4th-rank children) and social facial expression**

| Source of variance | *SS* | *df* | *MS* | *F* | *p* | $\eta^2_p$ |
|---|---|---|---|---|---|---|
| **Social status (between)** | 81.537 | 1 | 81.537 | 6.415* | 0.019 | 0.226 |
| **Error (between)** | 279.626 | 22 | 12.710 | | | |
| **Social facial expression (within)** | 18.426 | 1 | 18.426 | 5.649* | 0.027 | 0.204 |
| **Social rank* social dominance face** | 50.266 | 1 | 50.266 | 15.411** | 0.001 | 0.412 |
| **Error (within)** | 71.760 | 22 | 3.262 | | | |

* $p < 0.05$, **$p < 0.01$. *Note. The FN400 amplitudes of each facial expression were corrected by subtracting the averaged amplitude of the neutral facial condition.*

**Supplementary Table 4. FN400 comparisons between 1st- and 4th-rank children when they were evaluating**

**different social facial expressions**

| Social status | Face | *Mean* | *SD* | *t* | *df* | *p* | *Effect size (d)* |
|---|---|---|---|---|---|---|---|
| **Rank 1 vs. rank 4** | **DF** | 4.653 | 1.145 | 4.065** | 22 | 0.001 | 1.659 |
| **Rank 1 vs. rank 4** | **SF** | 0.560 | 1.163 | 0.482 | 22 | 0.635 | 0.197 |
| **Rank 1** | **DF vs. SF** | -3.286 | 2.480 | -4.589** | 11 | 0.001 | 1.223 |
| **Rank 4** | **DF vs. SF** | 0.808 | 2.626 | 1.065 | 11 | 0.310 | 0.273 |

**$p < 0.01$. *Note.* DF = dominant face, SF = subordinate face. *The amplitudes of FN400 were corrected by subtracting the averaged amplitude of the neutral facial condition.*

**Supplementary Table 5. Primer used for quantitative PCR**

| Gene | Forward primer (5’-3’) | Reverse primer (5’-3’) |
|---|---|---|
| *Bdnf* | *GGCTGACACTTTTGAGCACGTC* | *CTCCAAAGGCACTTGACTGCTG* |
| *Camk2* | *ACCCTGGCCTGGTCCTTCAATG* | *AGCCATCCTCACCACTATGCTGG* |
| *Cdk5* | *GGCTAAAAACCGGGAAACTC* | *CCATTGCAGCTGTCGAAATA* |
| *Creb* | *TCAGGGTACTACCATTC* | *TTCAGCAGGCTGTGTAGGAA* |
| *C-fos* | *TTCCTGGCAATAGCGTGTTC* | *TTCAGACCACCTCGACAATG* |
| *Egr1* | *CGAGCGAACAACCCTATGAG* | *CATTATTCAGAGCGATGTCAGAAA* |
| *Gria1* | *TTTTCTAGGTGCGGTTGTGG* | *CCT TTGGAGAACTGGGAACA* |
| *Gria2* | *AAGGAGGAAAGGGAAACGAG* | *CCGAAGTGGAAAACTGAACC* |
| *Npas4* | *GCTATACTCAGAAGGTCCAGAAGGC* | *TCAGAGAATGAGGGTAGCACAGC* |
| *GRIN2B* | *TCTGCCTTCTTAGAGCCATTCAG* | *AGACAGCTACAGCAGAGAC* |
| *Phf2* | *TGCCCGAACTGCGAGAAAACCC* | *TTTCACGTCCGGTGTTGGCCC* |
| *Ntrk2* | *GTGGTGTCATTAGTAGGTTCTTTGTT TT* | *ACTGAACCTGACCGTACAGAGTT TGGGTCTTTGCTGCC* |
| *Gapdh* | *GGCAAATTCAACGGCACAGT* | *GGGTCTCGCTCCTGGAAGAT* |
